# Using Museum collections to assess the impact of industrialization on mussel (*Mytilus edulis*) calcification

**DOI:** 10.1371/journal.pone.0301874

**Published:** 2024-04-17

**Authors:** Leanne A. Melbourne, Nathalie F. Goodkin

**Affiliations:** Department of Earth and Planetary Sciences, American Museum of Natural History, New York, New York, United States of America; Bigelow Laboratory for Ocean Sciences, UNITED STATES

## Abstract

*Mytilus edulis* is a commercially and ecologically important species found along the east coast of the United States. Ecologically, *M*. *edulis* improves water quality through filtration feeding and provides habitat formation and coastal protection through reef formation. Like many marine calcifiers, ocean warming, and acidification are a growing threat to these organisms—impacting their morphology and function. Museum collections are useful in assessing long-term environmental impacts on organisms in a natural multi-stressor environment, where acclimation and adaptation can be considered. Using the American Museum of Natural History collections ranging from the early 1900s until now, we show that shell porosity changes through time. Shells collected today are significantly more porous than shells collected in the 1960s and, at some sites, than shells collected from the early 1900s. The disparity between porosity changes matches well with the warming that occurred over the last 130 years in the north Atlantic suggesting that warming is causing porosity changes. However, more work is required to discern local environmental impacts and to fully identify porosity drivers. Since, porosity is known to affect structural integrity, porosity increasing through time could have negative consequences for mussel reef structural integrity and hence habitat formation and storm defenses.

## Introduction

Coastal ecosystems make up 10% of the world’s ocean systems yet host 90% of all marine life. Many calcifying organisms, like mussels, create three dimensional structures providing habitats that support high levels of marine biodiversity. Additionally, mussels and other bivalve mollusks provide additional ecosystem services in the form of water filtration and coastal protection [1,2]. Mussel and mussel reef ecosystem services are, however, dependent on robust shell formation.

Anthropogenic environmental changes are threatening all calcifying organism’s ability to form exoskeletons, as summarized in Cooley, Schoeman (3). Significant environmental changes expected by the end of the century including increased temperature and decreasing carbonate saturation (increased oceanic CO_2_) [3]. In laboratory experiments, increased CO_2_ conditions have led to weakened structural integrities through reduced growth, shell thinning and increased disorder in the calcium carbonate crystals in bivalve adult and larval shells, as summarized in Byrne and Fitzer [4]. However, most studies suggest that warming will have a bigger impact than acidification [5–7]. Warming waters will also lead to an increase in tropical cyclone frequency and intensification along the north east coast of the United States [8], leading to more intense pressure, through increased drag and lift forces, on these ecosystem engineers. Increasing our understanding of the environmental impacts on mollusks and mollusk reefs will be critical to understanding future risks.

Culture experiments investigating environmental impacts on calcification return contradictory results to experiments carried out using natural environmental gradients [9]. For example, laboratory experiments have shown negative impacts on calcification for corals and mollusks [10,11], whereas corals and mollusks transplanted along a natural carbonate saturation gradient calcify and grow at faster rates as pH levels fall [7]. Even laboratory experiment duration can alter the response to climate change indicating acclimation and/or adaptation ability. For example, the structural integrity of coralline algae under short term experiments (3 months) weakens in response to increasing CO_2_ concentrations [12], whereas in long term experiments (6 months) structural integrity is sustained highlighting acclimation potential. For the cold water coral, *Lophelia pertusa* (syn. *Desmophyllum pertusum*), negative calcification occurs under short term high CO_2_ experiments, while calcification rates are enhanced in long term experiments (6 months) [13]. This highlights the importance of acclimation and/or adaptation potential in responses to climate change.

Museum collections add a new dimension to these studies allowing a natural study of how multiple stressors in a natural environment impact a species through time. Collections also allow us to examine the long-term impacts of environmental change whilst accounting for the ability of organisms to acclimate and adapt. For example short-term, laboratory experiments on coralline algae reveal carbonate driven changes to internal cellular structure weakening structural integrity [12]. However, utilizing museum collections show that the internal cellular and structural changes over the last 130 years are not as large as laboratory experiments imply [14], indicating acclimation potential, which is also seen in long term studies [15]. Long-term mussel studies are less clear. For example, samples collected in the early 2000s from the United States (US) west coast of *Mytilus californius* have thinner shells than samples collected from two Native American midden sites (1000–2420 years BP) and the 1960s–1970s [16]. Whereas shells of *Mytilus edulis* from the Belgian coastline thicken through time (1906–2016) [17]. These examples show that field experiments and long-term time studies using museum collections and fossil material highlight that reactions to environmental change are not as clear as laboratory experiments suggest.

The American Museum of Natural History (AMNH) has large bivalve collections that go back to the late 1800s/ early 1900s from the northeastern US, an ideal location to evaluate environmental change. The coastal part of the northeastern US has experienced over 2°C increase in temperatures on land since 1902 [18]. Since the early 1980s, the continental shelf waters between Cape Hatteras, North Carolina and Cape Chidley, Newfoundland has experienced increases in temperature of 0.37 ± 0.06°C/decade, which is similar to changes of 0.39 ± 0.06°C/decade on the continental slope between North Carolina and Labrador, Canada [19]. Longer term coarser datasets (1900–2018) show an increase in sea surface temperature (SST) of 0.10 ± 0.01°C/decade [19]. Additionally, over the last 150 years, ocean surface waters have become 30% more acidic due to the dissolution of anthropogenic CO_2_ [20]. Coastal waters have the added influence of the local environment including nutrient input, freshwater loading and the role of primary producers in changing carbonate chemistry [21,22] that leads to higher variability of CO_2_ dissolution than the open ocean [23]. For the northeast coast of the US, the freshwater input has led to a lower buffering capacity that potentially makes the northeast coast more susceptible to acidification than the south coast [24]. While, the proportion of very intense storms and the frequency with which they rapidly intensify has also increased over the last 40 years [25].

By assessing mussel structural integrity over the last 130 years, we can make informed predictions on if structural integrity will be impacted in the future and if ecosystem function will be sustained under a more turbulent environment. In this study, we focus on how mussel shape, shell thickness and shell density (all parameters that affect structural integrity) have changed through time (pre-industrialization to now) along the east coast of the US to infer how ecosystem function may be impacted and predict how this may change in the future.

## Methods

### Specimen collection

*Mytilus edulis* shells were collected from the intertidal zone in a variety of localities from four US State coastal economic zones: Massachusetts, Connecticut, New Jersey, and New York. Up to 10 individual valves from each locality were collected. A permit for scientific collection, issued by the Commonwealth of Massachusetts, Division of Marine Fisheries, was obtained for the modern samples collected in Massachusetts, as these were the only live specimens collected. Modern samples were geographically paired with historical shells within the collections of the AMNH. Modern specimens were cleaned and left to air dry overnight. Shells were collected from three age periods early historical (1890–1915), mid historical (1961–1963) and modern (2021–2023) (Table 1).

**Table 1:** Mussel specimen information. Summary of specimens used in the study with information on site, locality, collector, and date collected. Numbers next to locality correlate with numbers in Fig 1.

Site	Locality	State	Year	Collector	Date Collected	Number of valves
**1**	Nahant, Essex County (a)	Massachusetts	Early Historical	Coll. Unknown	1911	10
**1**	Canoe Beach, Nahant Bay (b)	Massachusetts	Modern	Ross Ong and Leanne Melbourne	2023	30
**2**	Vineyard Haven (c)	Massachusetts	Early Historical	Coll. Unknown	1910	5
**2**	Woods Hole (d)	Massachusetts	Early Historical	R W Miner	1912	4
**2**	Grassy Island, Vineyard Sound (e)	Massachusetts	Modern	Natalie Umling	2022	4
**2**	Lake Tashmoo, Vineyard Sound (f)	Massachusetts	Modern	Ross Ong and Leanne Melbourne	2023	22
**2**	Lambeth’s cove, Vineyard Sound (g)	Massachusetts	Modern	Ross Ong and Leanne Melbourne	2023	13
**2**	Menemsha Basin, Vineyard Sound (h)	Massachusetts	Modern	Ross Ong and Leanne Melbourne	2023	30
**3**	Orient Point, Long Island (i)	New York	Mid Historical	Alice Denison Barlow	1963	4
**3**	Montauk, Long Island (j)	New York	Modern	Nathalie Goodkin	2021	8
**4**	Sherwood Island (k)	Connecticut	Mid Historical	D. Germer	1962	10
**4**	Sherwood Island (l)	Connecticut	Modern	Leanne Melbourne	2022	9
**4**	Bayville, Long Island (m)	New York	Mid Historical	W. Old Jr	1961	10
**4**	Westchester, Rye Beach (n)	New York	Early Historical	D.M. Fisk	1913	4
**4**	City Island, Bronx County (o)	New York	Early Historical	F Kessler	1915	4
**4**	Orchard Beach (p)	New York	Mid Historical	G.Eddison	1963	10
**4**	Orchard Beach (q)	New York	Modern	MAT residents	2022	7
**5**	Sheepshead Bay (r)	New York	Early Historical	R.E.Willinger-William S.	1890	10
**5**	Far Rockaway (s)	New York	Early Historical	Coll. Unknown	1909	6
**5**	Coney Island Beach (t)	New York	Modern	Natalie Umling and Leanne Melbourne	2021	10
**5**	Brighton Beach (u)	New York	Modern	Natalie Umling and Leanne Melbourne	2021	10
**5**	Jacob Riis Park, Far Rockaway (v)	New York	Modern	Natalie Umling and Leanne Melbourne	2021	10
**5**	Raritan Bay (w)	New Jersey	Mid Historical	Coll. Unknown	1961	8
**5**	North Beach, Sandy Hook (x)	New Jersey	Modern	Leanne Melbourne	2022	10

### Study sites

**Fig 1:**
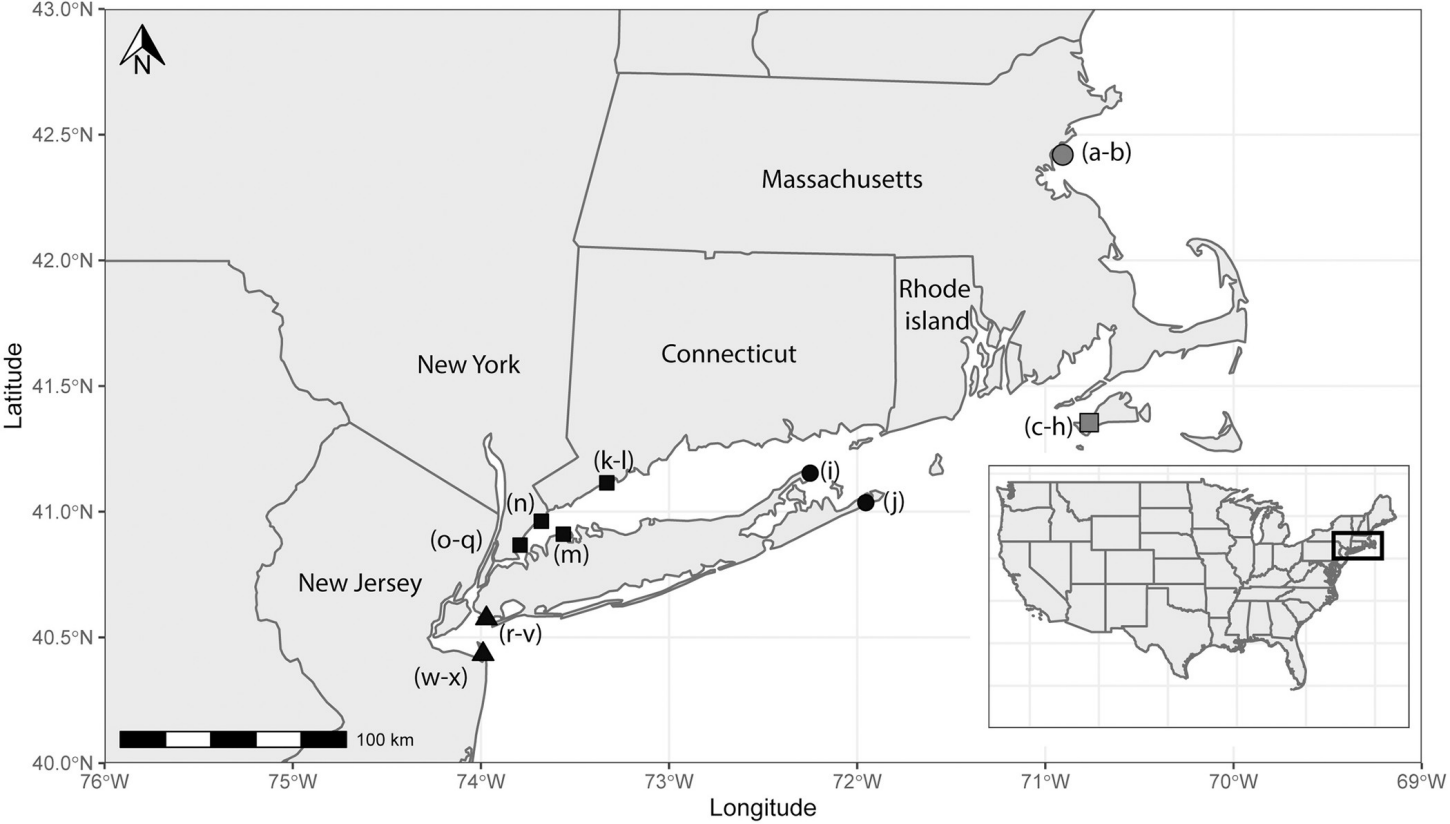
Site and locality map. Map derived from the rnaturealearth package in RStudio (version 2021.09.0) showing collection locations indicated by shapes and letters. Shapes define ‘sites’ used in analysis. Site 1 (grey circle), Site 2 (grey square), Site 3 (black circle), Site 4 (black square) and Site 5 (black triangle), while letters define the site localities. Details can be found in Table 1.

**Fig 2:**
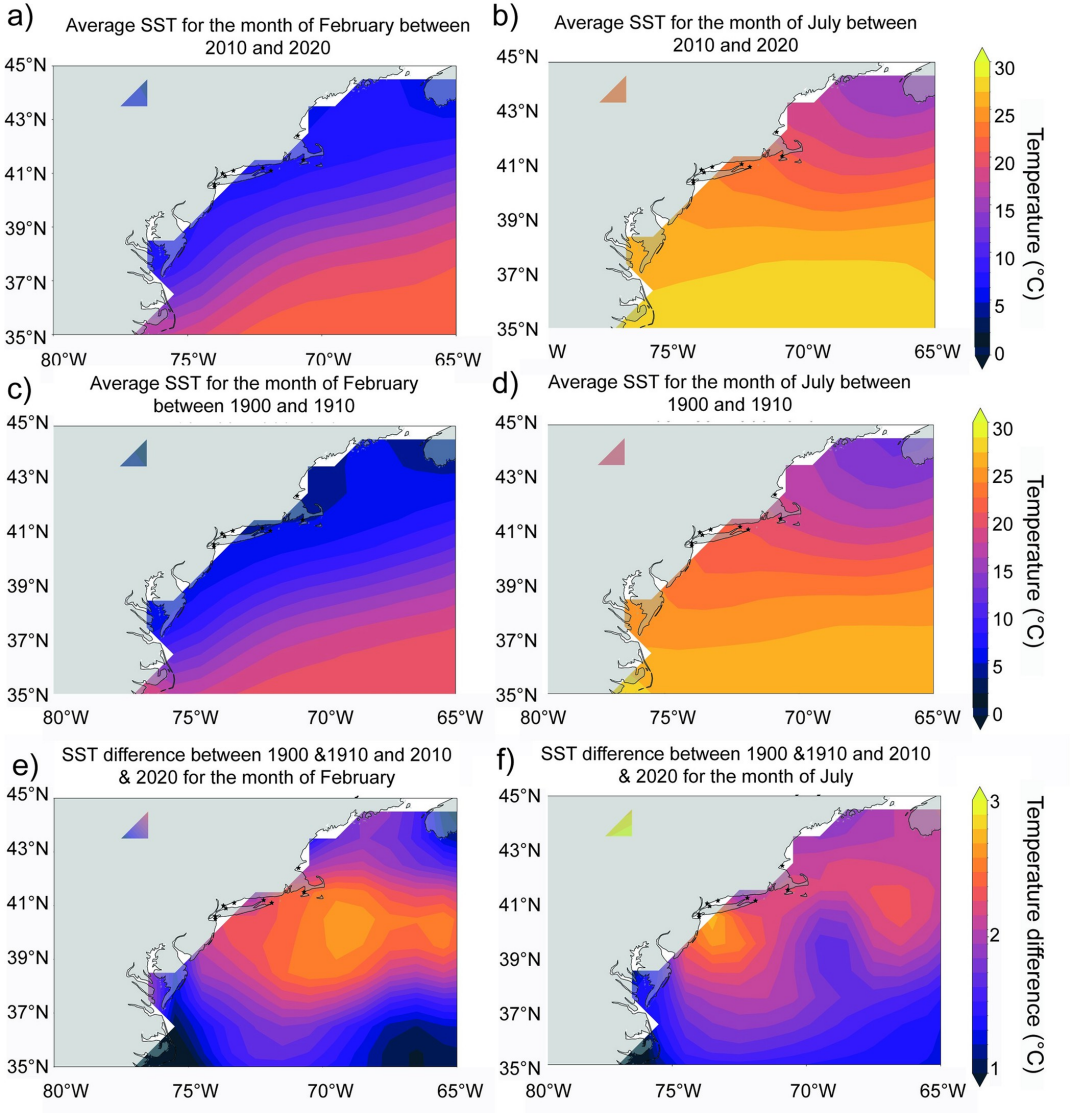
Spatial sea surface temperature maps. Reanalysis sea surface temperature (SST) for a) February and b) July 2010–2020, for c) February and d) July and for the difference between 2010–2020 and 1900–1910 for e) February and f) July.

**Fig 3:**
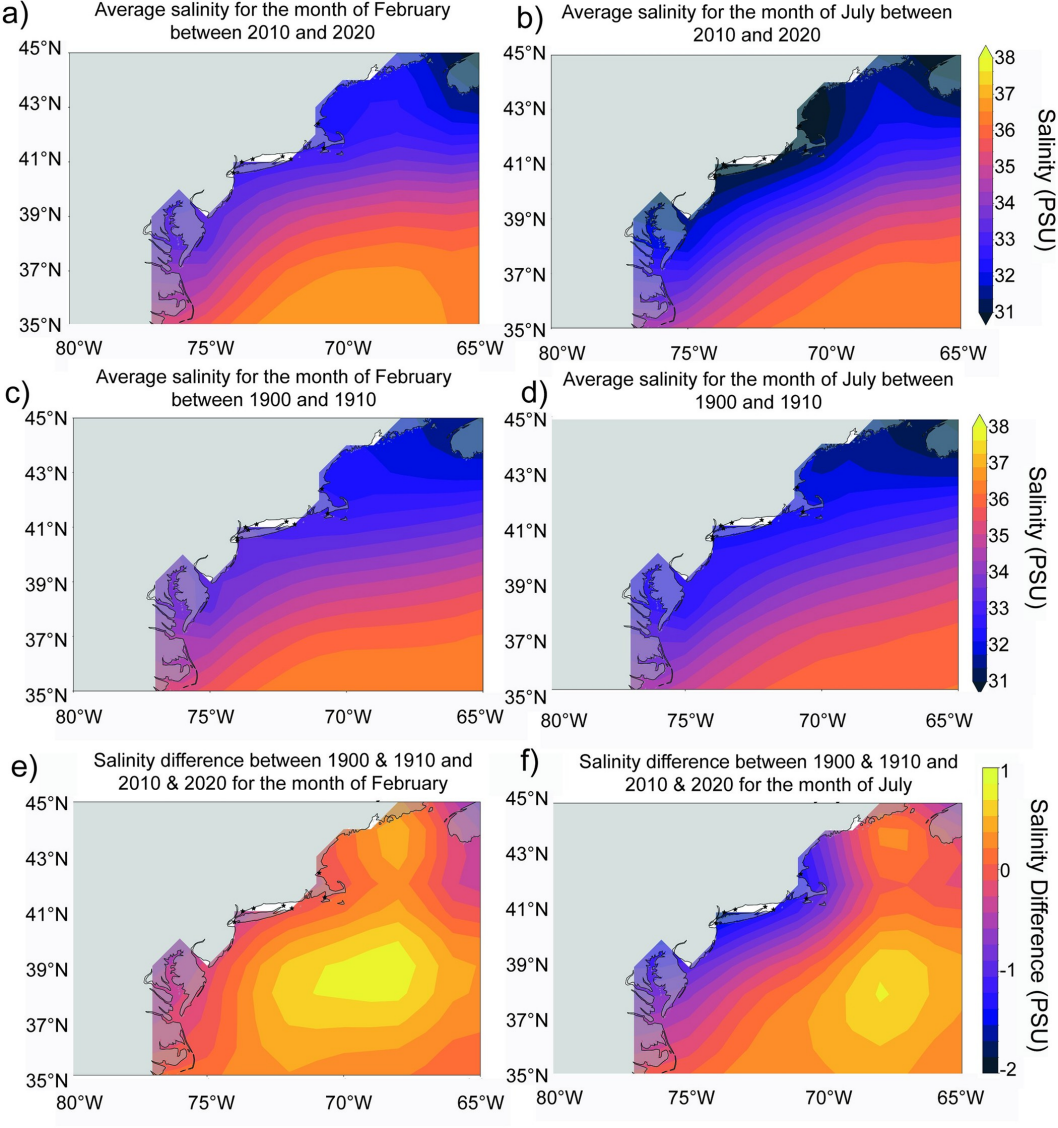
Spatial sea surface salinity maps. Reanalysis for sea surface salinity (SSS) for a) February and b) July averaged from 2010–2020, for c) February and d) July averaged from 1900–1910, and the difference between 2010–2020 and 1900–1910 for e) February and f) July.

Mussels grow in coastal saline to brackish waters. Localities have been grouped into 5 sites based on similar water basins and geographical proximity (Fig 1). The sites are Nahant Bay (Site 1), the southern end of Cape Cod (Site 2), the tip of the Long Island Sound (Site 3), western Long Island Sound (Site 4) and New York Harbor (Site 5). In present time, each location experiences a large seasonal range (5–25°C) of SST with minima in February and maxima in July (Fig 2A and 2B) [26]. Seasonal sea surface salinity (SSS) variability is different between the northern and southern sites, with the southern sites ranging from 31–34 PSU, and the northern sites remaining below 33 PSU (Fig 3A and 3B) [27]. Seasonality is based on reanalysis data and may not represent the full range of mussel growth at the more coastal locations; however, the changes are likely to be on the same magnitude as regional changes.

Reanalysis data also shows significant changes to SST [26] and SSS [27] over the past century when SST has increased between 1 and 3°C in the mid-Atlantic bight (Fig 2). Although warming was not uniform, with the most prominent warming occurring in the most northern sites in the winter (2°C), while in the most southern sites the most prominent warming occurred in the summer months (3°C) (Fig 2E–2F). For SSS, the change since the 1900s was more uniform between sites with a freshening of 1 PSU in the summer months and a little to no change in the winter (Fig 3E–3F).

### Shell shape

All valves were measured for length (maximum distance on the anterior-posterior axis), height (maximum distance on the dorsal-ventral axis, perpendicular to the length) and width (maximum distance on the lateral axis) using digital calipers (± 0.05 mm) (S1 Fig).

To measure surface area, the right valves were scanned using the GE phoenix v|tome|x s240 computerized tomographer (CT) with various parameters (current, voltage, voxel size, type of filter and type of ray detector used) to optimize the contrast and resolution of the x-ray images (S1 Table). The valves were wrapped in aluminum foil. Shell surface area and volume were calculated from reconstructed volumes using VG studio (v 5).

A geometric morphometrics approach, based on Telesca, Michalek [28], was used to analyze *Mytilus* shell shape. An elliptic Fourier analysis (EFA) of outlines was used to examine shell shape variation between locations and through time. Orthogonal lateral and ventral views of CT scanned shells were taken using 3D slicer. Images were then centered, aligned, and converted into black masks on a white background (8-bit, grey-scale mode,.jpeg format with no level of compression) in ImageJ. Images were then imported into RStudio (version 2021.09.0) using the momocs package [29], outlines of intact shells were used in the analysis. Isolated outlines were converted into a list of x-,y- coordinates. Lateral and ventral views of each shell were separated and processed independently. Extracted outlines were then visually inspected to assess size, rotation, and alignment differences. Outlines were smoothed, centered, and scaled. 1000 pseudo-marks were sampled along each outline and point configurations were aligned through a Procrustes superimposition and starting points normalized. After analyses 10 harmonics were chosen and four coefficients per harmonic (40 descriptors) were extracted for each outline and used as variables quantifying the geometrical information.

A principal component analysis (PCA) was then performed on the Fourier coefficients to observe shape variation between sites and through time. Principal components (PCs) were calculated to define new axes that captured the most shape variation among individuals. The first two principal component scores (PC1 and PC2), derived from the morphometric analysis, were used to plot an empirical morphospace. Code is provided in the (S1 File).

### Shell thickness

The left valves were embedded in epoxy resin and cut parallel to the long axis of growth through the umbo to expose the internal shell using a Buehler low speed saw. These thick sections were then polished through a series of silicon carbide papers (P400-P1200). The embedded shell valves were used to measure shell thickness. A minimum of ten measurements on each shell were made based on light microscope images. Measurements included overall shell thickness, as well as measurements of the calcite and aragonite band individually, which were identified by changes in color under light microscopy. From these measurements the average thickness, maximum thickness and percentage calcite were calculated. Both average thickness and maximum thickness were divided by the length of the specimen to incorporate the influence of size on thickness.

### Shell density

Apparent porosity, bulk density and micro-density were measured using a modified version of the buoyant weight method [30], employing a density kit for XPE/XS analytical balances Mettler Toledo (± 0.1 mg; Mettler Toledo., Columbus, OH, USA). Apparent porosity here refers to the percentage of the pore volume connected to the external surface, bulk density is the mass per unit volume of the shell material and the volume of pores, while micro-density is the mass per unit volume of just the shell material.

Dry shell weight (**DW**) was measured three times using an analytical balance (± 0.1 mg). Shells (right valves) were placed in a desiccator connected to a mechanical vacuum pump for about 1 h to remove all water and air from the pores. Under vacuum conditions, the dry shells were soaked by gradually pouring distilled water inside the desiccator. A three-way tap was added to the desiccator to be able to add water slowly while still under vacuum conditions. Once fully saturated, shells were taken out of the water, quickly blotted to remove surface water, and weighed in the air three times, making sure that the weighing platform was completely dry to establish saturated mass of the shell or mass of the shell plus mass of the water enclosed in its pores (**SW**), ensuring that no air bubbles adhered to its surface. The fully water saturated shell was slowly lowered onto the underwater weighing pan and was weighed three times to establish the buoyant mass of the shell or mass of the shell fully saturated with water minus mass of the water displaced by it (**BW**).

Parameters calculated from DW, SW and BW where ρ density of the fluid medium (in this case, distilled water) was 0.99823 g cm^−3^ at 20°C and 1 atm:

V_BIOMINERAL_−the volume of mineral shell, excluding the volume of its pores

=(DW–BW)/ρ (1)

V_PORES_−volume of the pores in the shell

=(SW–DW)/ρ (2)

V_TOT_−total volume of the shell including its pores

$$=V_{BIOMINERAL}+V_{PORES}$$ (3)

Additionally, the following skeletal parameters were calculated:

$$Micro-density=DW/V_{BIOMINERAL}$$ (4)

$$Bulk-density=DW/V_{TOT}$$ (5)

$$Porosity=(V_{PORES}/V_{TOT})\;x\;100$$ (6)

Two shells from sites with the highest and lowest porosities were re-embedded and polished through a series of silicon carbide papers (P400-P1200) and aluminum oxide (5 μm and 1 μm). The polished specimens were then carbon coated and analyzed under Scanning Electron Microscopy (SEM) to assess for visible differences in porosity. The number and size of visible pores was assessed qualitatively between SEM images of samples from a high and low porosity site.

### Statistics

Statistics were calculated in RStudio (version 2021.09.0). Data was first checked for normality using the QQP (quantile -quantile plot) function. Data fitted a normal distribution. For assessing differences between locations and time periods, a mixed effects model was used with sites and year as fixed effects and individuals classed as random effects. A pairwise t-test was used to assess which sites within time periods and which time periods within sites were significantly independent (S2 File). For the mixed effects model lme4 package [31] was used and the emmeans package [32] was used for the post hoc tests. A PCA was performed on the variables: length, width, height, surface area to volume ratio (SAV), micro density and apparent porosity. For the Elliptical Fourier Analysis PCA, the PCs were analyzed with a multivariate analysis of variance (MANOVA) to test for significant effects between sites and through time on shape variances (S3 File). All code can be found in the (S2 and S3 Files).

## Results

### Shell density and thickness

**Fig 4:**
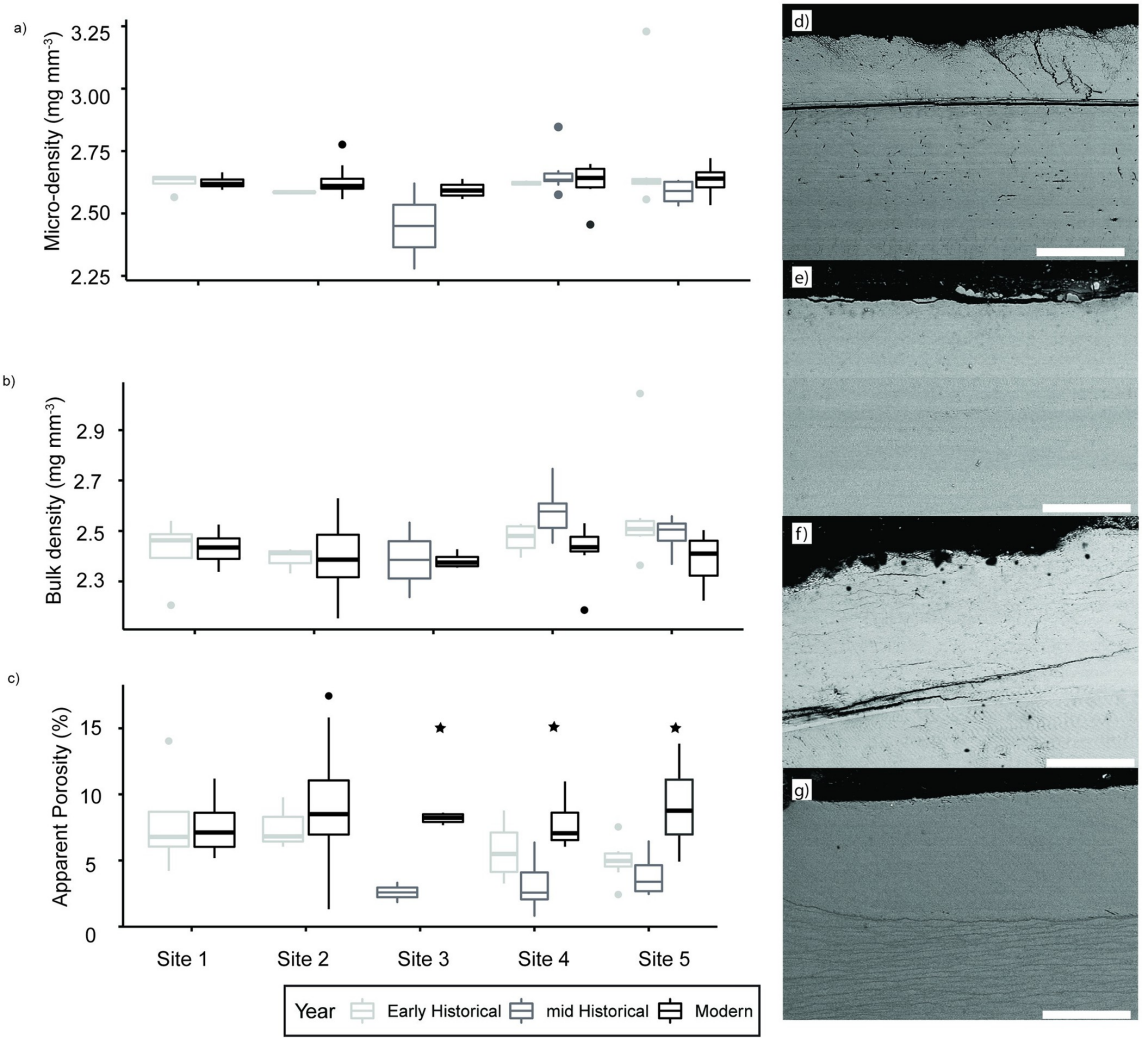
Density plots. Box and whisker plots showing the micro density (a), bulk density (b) and apparent porosity (c) separated by year and site. Black stars denote significant differences. Backscatter electron images of low porosity mussel shells from site 4 in 1961 (d&e) and high porosity mussel shells from site 5 in 2021 (f&g). Scale bar 100 μm.

Only apparent porosity was significantly different through time (F_2_,_165_ = 14.107, p<0.005) (Fig 4A–4C, S2 Table). Modern samples were significantly more porous than mid-historical material in sites 3, 4 and 5. Modern samples were significantly more porous than early historical material only in site 5. Sites 1 and 2 showed no significant changes in porosity through time. At Sites 4 and 5, the mid-historical material had lower porosities than the early historical material, although these results were not significant. All shell thickness parameters were significantly different between sites but not through time (pcal: F_4,93_ = 27.934, p<0.005; average thickness: F_4,93_ = 12.682, p<0.005 and maximum thickness: F_4,93_ = 12.682, p = 0.05). Focusing on modern material, Site 2 had a significantly higher percentage of calcite than site 1, 4 and 5. Site 4 had significantly larger average and maximum thicknesses than sites 2 and 5 (Tables 2 and S2).

**Table 2:** Mussel shell measurements. List of density (micro density, bulk density, and apparent porosity), morphological (length: Height ratio, length: Width ratio and surface area: Volume ratio), and thickness (% calcite, average thickness/ length and maximum thickness/ length) measurements for each site and time period.

Site	Year	Micro-density (mg/ mm^-3^)	Bulk density (mg/ mm^-3^)	Porosity (%)	Length (mm)/ Height (mm)	Length (mm)/ Weight (mm)	Surface Area (mm^2^)/ Volume (mm^3^)	% calcite	Average thickness (mm)/ Length (mm)	Maximum Thickness (mm)/ Length (mm)
**1**	Early	2.63 ± 0.02	2.42 ± 0.07	7.95 ± 2.12	1.98 ± 0.04	4.79 ± 0.24	3.89 ± 0.59	47.29 ± 2.50	0.025 ± 0.003	0.030 ± 0.003
	Modern	2.62 ± 0.003	2.44 ± 0.01	7.13 ± 0.34	1.93 ± 0.02	4.55 ± 0.05	1.73 ± 0.22	48.75 ± 1.89	0.022 ± 0.004	0.032 ± 0.005
**2**	Early	2.59 ± 0.002	2.39 ± 0.03	7.55 ± 1.13	1.84 ± 0.02	5.26 ± 0.19	4.07 ± 0.54	57.75 ± 1.31	0.015 ± 0.002	0.021 ± 0.002
	Modern	2.62 ± 0.004	2.41 ± 0.01	8.28 ± 0.44	1.91 ± 0.01	5.21 ± 0.03	3.58 ± 0.31	63.76 ± 1.69	0.016 ± 0.001	0.022 ± 0.002
**3**	Mid	2.45 ± 0.17	2.39 ± 0.15	2.59 ± 0.73	2.11 ± 0.03	5.51 ± 0.07	2.60 ± 0.08	45.33 ± 1.88	0.017 ± 0.001	0.026 ± 0.005
	Modern	2.60 ± 0.02	2.38 ± 0.02	8.18 ± 0.21	1.89 ± 0.04	4.83 ± 0.25	3.82 ± 0.87	56.26 ± 4.33	0.017 ± 0.003	0.025 ± 0.005
**4**	Early	2.62 ± 0.004	2.47 ± 0.03	5.76 ± 1.22	1.88 ± 0.07	5.28 ± 0.20	4.13 ± 0.62	53.26 ± 2.39	0.018 ± 0.001	0.024 ± 0.003
	Mid	2.65 ± 0.02	2.57 ± 0.02	3.16 ± 0.49	2.07 ± 0.02	4.82 ± 0.08	2.53 ± 0.12	46.90 ± 1.71	0.023 ± 0.002	0.033 ± 0.002
	Modern	2.62 ± 0.03	2.42 ± 0.04	7.77 ± 0.67	2.00 ± 0.04	4.77 ± 0.13	2.59 ± 0.26	42.46 ± 5.51	0.025 ± 0.002	0.036 ± 0.002
**5**	Early	2.69 ± 0.08	2.56 ± 0.07	4.98 ± 0.51	1.97 ± 0.02	5.48 ± 0.08	3.49 ± 0.17	52.18 ± 2.68	0.014 ± 0.001	0.020 ± 0.001
	Mid	2.59 ± 0.02	2.48 ± 0.04	3.92 ± 0.91	1.84 ± 0.02	5.16 ± 0.1	4.73 ± 0.34	61.59 ± 5.27	0.017 ± 0.001	0.022 ± 0.002
	Modern	2.63 ± 0.01	2.39 ± 0.02	9.27 ± 0.62	1.95 ± 0.02	5.16 ± 0.07	3.93 ± 0.21	53.40 ± 1.53	0.016 ± 0.001	0.024 ± 0.001

SEM images of samples from low porosity and high porosity sites show that porosity is mainly constrained to the calcite layer. Variation in porosity between samples within the same sites were very large (Fig 4D–4G), therefore, within the small subset, we were unable to discern porosity differences between sites visually.

### Shell shape

Both length: height and length: weight ratios were not significantly different through time. Length: width ratios were significantly different between sites (F_4,338_ = 10.763, p<0.05) (Table 2). Focusing on just modern material, Site 1 had significantly smaller length to width ratios than sites 2 and 5, while site 2 had significantly larger length to width ratio than Site 4. There were no significant differences between sites or through time for surface area: volume ratios (Tables 2 and S2).

**Fig 5:**
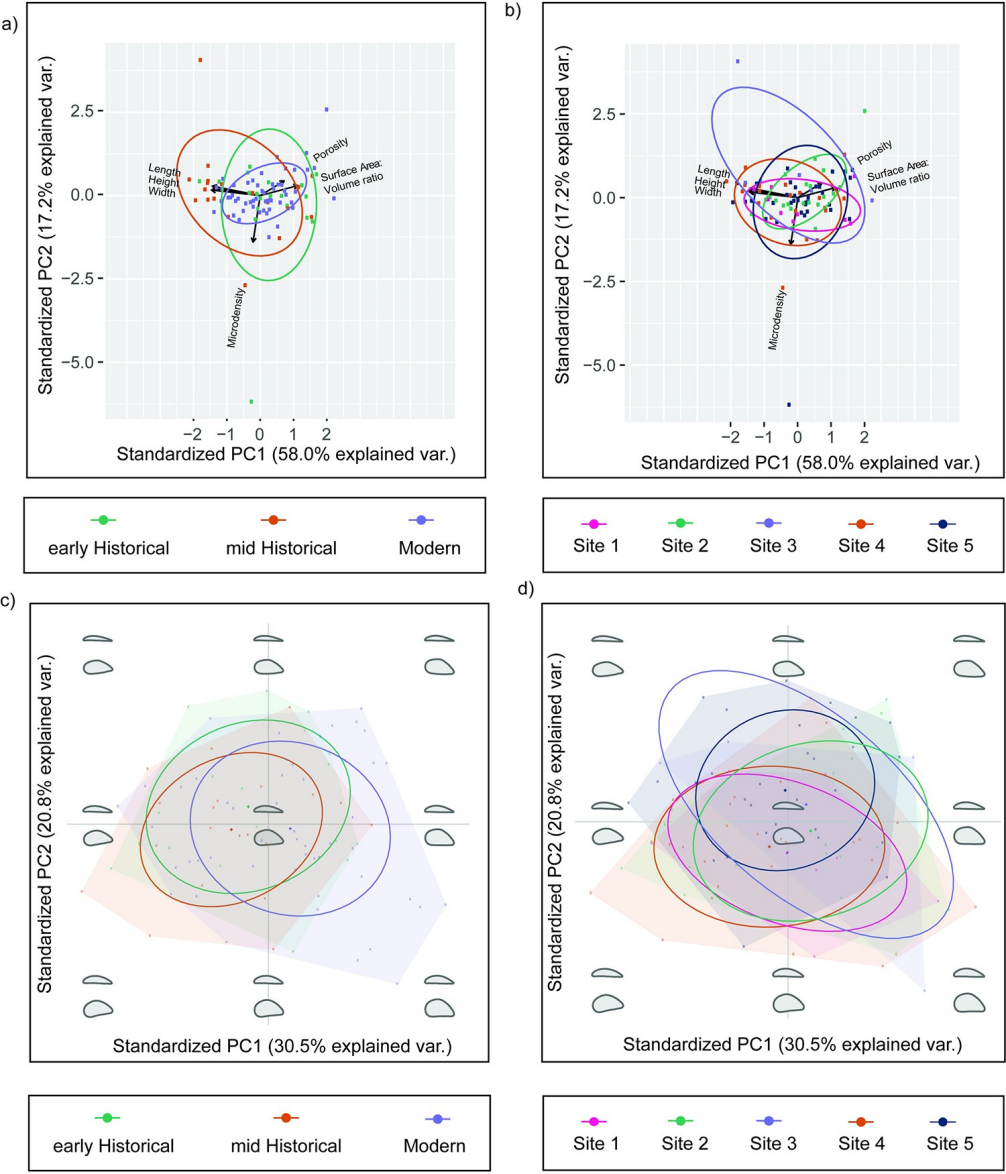
Morphometric analysis plots. PCA plots based on measured parameters evaluated by time (a) and by site (b). Elliptical Fourier analysis on morphological shape by time (c) and by site (d).

For the PCA that focused on variables length, width, height, Surface Area: Volume ratio (SAV), micro density and apparent porosity (Fig 5A and 5B), the first 10 PCs accounted for 92% of variability. PC1 accounted for 58.0% variation, while PC2 accounted for 17.2% variation. The PCA plots show that length, width, height, porosity, and SAV contribute to PC1 while micro-density contributes to PC2. Examining the PCA with respect to time, all three time periods overlap, however early historical and mid historical groups exhibit more variation along the PC2 axis, whereas the modern group is more constrained along PC2 (Fig 5A). Geographically, sites 1,2,4 and 5 appear to occupy similar positions in the morphospace spread along the PC1 axis, whereas Site 3 appears to be more variable in PC2 than the other sites (Fig 5B).

In the Elliptical Fourier Analysis (Fig 5C and 5D), 91.7% of the variation was accounted for in the first 10 PC axes, while 67.1% of the variation was accounted for in the first 3 PC axes. PC1 accounts for 30.5% of the variation and relates to the shell height. Low values represent narrow (short) in height shells, whereas high values represent wide (tall) in height shells (S2 Fig). PC2 (20.8% of the variation) relates to the shell width and the dorsal-ventral shape. Low values represent a wide shell width with a concave shape that gradually transitions to a more elongated, convex shape and a narrow shell width (S2 Fig). MANOVAs revealed significant effects on year (Wilk’s λ = 0.44, approx. F_2,180_ = 3.72, p < 0.001) and between sites (Wilk’s λ = 0.33, approx. F_4,180_ = 2.38, p < 0.01) on mussel shape variation. Focusing on the PCA plots separated by year, the early- and mid-historical groups have similar spreads over the PC1 and PC2 axis, however, are slightly offset from each other. The early-historical extends into a space with more elongated, convex narrow shells, while mid-historical extends into the space with wider concave shells. The modern group is more variable across PC1 and less variable in PC2 than the other two groups leading to the group offsetting into a new space which is wider in shell width and height and more concave shape. Sites 1, 2 and 4 occupy the same space which sees more variation in PC1 axes (the full range in shell height) and little variation in PC2 axes. Site 5 slightly extends into a space with narrower convex shells. While Site 3 has more variability by encompassing across the top left corner (convex shells that are narrow in both height and width) and bottom right corner (concave shells that are wider in both height and width).

## Discussion

Environmental change is affecting mussel populations along the northeast coast. Our results show that climate change over the last 130 years, at certain locations, coincides with an increase in porosity in *M*. *edulis* shells (Fig 4). In other reef formers, such as corals, porosity changes are known to lead to weakened structural integrity [33,34], therefore the increased porosity seen in mussels through time may have a negative impact on ecosystem function. As weakened structural integrity could lead to increased predation on mussels, as shells are easier to break, but also increase susceptibility to wave erosion. With the projections of increased tropical cyclones in the Atlantic [8], mussel ecosystem function may be challenged due to increasing porosity leading to decreased habitat complexity [35]. It is important to note that the measured porosity changes are quite small and have yet to impact overall bulk density, but if porosity continues to increase in the future due to environmental change this may negatively affect ecosystem function.

There have been very few studies on porosity in bivalves, limiting our understanding of what is driving the changes. Gizzi, Caccia (30) showed that changes in the porosity of clams (*Chamelea gallina*) coincided with solar irradiance and temperature, where warmer temperature localities had more porous shells. The authors suggest that the warmer, more irradiated populations consume more energy for growth which reduces the amount of energy available for shell formation. As temperatures have increased over the last 150 years [19], and reanalysis data indicates a temperature increase of 1–3°C between 1900 and 2021 in the Mid Atlantic Bight (Fig 2), this could suggest that the increase in temperature has led to the increased porosity within mussel shells on the east coast. Especially as warming in the mid-Atlantic bight since the 1900s was not uniform and the largest change in temperature only impacted the growth season in Sites 3–5. This, therefore, might explain the lack of change in porosity at Sites 1 and 2.

The lower porosities seen in the mid historical also support a temperature influence on porosity. Focusing on a long-term time series of temperature anomalies over the northwest Atlantic, there is a decrease in temperature from the 1950s to the 1970s, and similar negative temperature anomalies in the early 1900s [19]. The drop in temperature coincides with increased sulfur aerosols from increased industrialization at the end of World War 2, which led to global temperatures decreasing into the 1970s [36]. Additionally, the North Atlantic Oscillation (NAO), a dominant influence over climate variability in the North Atlantic relates to shifting atmospheric air pressure between the Azores and Greenland, was in a negative phase [37]. This also meant colder, drier winters during this period. It has been shown that the ideal conditions manifested during a negative NAO led to increased bivalve landings [38], highlighting optimum conditions and therefore could explain the lower porosities at this time period.

We rule out dissolution and organic degradation as a reason for changes in porosity due to a lack of visible dissolution in SEM images of the most porous samples. Secondly, all modern material is statistically similar in porosity and our modern samples are composed of live-collected mussels, dead mussels still attached to the substrate and beach collected shells (all potential possibilities for our historical material). Finally, if the degradation of organic matter left behind pore space and therefore increasing porosity, it is more likely that we would see lower porosity in historical material, which we do not.

Interestingly, shell shape or shell thickness do not appear to be influenced by temperature. Studies on European mussels document the influence of increased temperature on growth, which includes increased mortality [5], increased metabolic rate [39], increased crystal disorder [40], as well as combined with salinity changes decreasing shape heterogeneity and increasing shell thickness [28,41]. The lack of variation in shell shape and thickness between early historical and modern material and between modern locations, in our results, suggests the mussels on the east coast of the US do not respond to temperature and salinity in the same way as European mussels. Hoppit and Schmidt [42]’s meta-analysis on European benthic organisms have shown the negative impacts of climate change on calcification in European mollusks, which contrasts global analysis, and therefore has led the authors to suggest that European mollusks may be more sensitive to climate change stressors than the global consensus. Therefore, our results may be an indication of European mollusks being more sensitive to temperature and salinity than their American counterparts. Or our results highlight a redirection of energy suggested in Gizzi, Caccia [30] in which increasing temperatures may have led to energy being redirected to maintain shape and thickness at the detriment of maintaining shell porosity.

The lack of change in shell thickness through time also suggests that changing carbonate chemistry at this setting is not affecting mussel calcification in the same way as seen in other studies. Bivalve growth rates are negatively impacted by climate stressors such as ocean acidification [43]. It has been shown that lowering the pH of the seawater influences pH regulation in the extra-pallial fluid of bivalves [44–46] and other physiological process like oxygen consumption [47,48] and increased metabolic rates [45]. Therefore the energy redirected to maintain calcification could be at the expense of shell formation and growth [44,45]. Aspects of shell formation altered by increased acidity include reduced shell growth [45,49] and size [50], increased structural disorder within crystals [49,51,52], alteration of material properties [4,52,53] and reduced shell thickness [4,16,54]. Increased porosity, due to ocean acidification, has not been seen in bivalves before but has been seen in many marine calcifiers including corals, tube worms, echinoderms, and coralline algae [4,12]. Therefore, if changing carbonate chemistry is altering growth here, it could be behind the porosity changes through time, while other parameters such as shell thickness are maintained.

Alternatively, our results may suggest that the changing carbonate chemistry along the east coast is not large enough to elicit a response in shell calcification. Transgenerational exposure to elevated pCO_2_ in bivalves has been shown to significantly alleviate the negative impacts of ocean acidification [55] and therefore the lack of change in shell thickness could be caused by the changing carbonate chemistry along the east coast being within the acclimation potential of the mussel. This is further supported by another study which found adult *M*. *edulis*, from Maine, to be sensitive to warming but tolerant to moderate acidification predicted for the end of the century [39].

Without detailed environmental information it is impossible to tease apart whether warming, ocean acidification or a combination of the two is behind porosity changes through time. Laboratory and mesocosm experiments have shown combined warming and acidification have led to decreases in somatic and shell growth [5], increases in calcified mass to soft tissue ratios [56] and decreased calcification rates [7]. The combination of warming and acidification may be impacting shell porosity, as energy is redirected to maintain growth, shape, and shell thickness against a warmer, more acidified environment. Although some studies have highlighted the competing impacts of warming and acidification, for example while crystal structure becomes more disordered under warming is it maintained under both warming and acidification [40]. Therefore, the warming and acidification occurring along the east coast of the US may be counteracting with each other leading to a lack of response in shell shape and thickness.

Again, detailed information of the habitat itself is also important. For example, increased eutrophication, food supply and water temp can buffer ocean acidification impacts leading to unusual responses of shell thickening through time [17]. Additionally, population density and growth rates alter shell morphology [57], while tidal heights/ wave exposure are known to influence growth rates [58,59]. For example, *M*. *galloprovincialis* had faster growth rates and narrower shells on exposed shores compared to sheltered shores [60]. Thicker shells were found at sheltered and the most exposed sites [60], which differs from other studies that found thinner shells at the sheltered sites [61]. It is thought that tidal heights/ wave exposure either alter predator interactions or food availability in the water column, which in turn affect growth rates and other parameters [1,62,63]. Leading Seed and Suchanek (1) to suggest that food availability may be the most important factor impacting growth. Increased food supply caused by increased nutrient enrichment has been found to positively correlate to abundance, biomass, and assimilation efficiency, whereas increased mortality, reduced biomass, and lower recruitment occurs when increased nutrient enrichment led to habitat loss or degradation [64]. Increased food supply can mitigate the negative effects of acidification on calcification, amplifying growth impacts [65]. Therefore, food availability, local nutrient variation and tidal data could be the reason for the significant differences between modern samples at different sites and the small deviations between sites in the PCA plots. A more detailed assessment of the local environment including nutrient information and tidal data at our five sites would help tease apart site differences.

Historical material in museum collections can be used to answer important questions about the impacts of environmental change on century timescales. Our results demonstrate a small but measurable increase in porosity at 3 of our 5 locations, coincident with rising SST particularly during the months of maximal growth at the three sites. Although, the lack of detailed information accompanying our historical material prevents us from fully explaining the overall higher porosities in the historical material at sites 1 and 2 compared to sites 3,4 and 5. Based on our work, additional studies could take the form of local monitoring of sites and/or culture experiments including varying temperature, carbonate concentrations, and/or food/nutrient availability to assess single and/or multiple drivers on porosity. This would help us to tease apart the different drivers and address the main driver of porosity changes through time in *M*. *edulis* along the east coast of the US and therefore predict how porosity might further change. In understanding the different drivers and how they interact with each other, we can then predict how structural integrity and ecosystem function within east coast mussels will be impacted under future climate change.

## Supporting information

S1 Fig Bivalve morphology. Visualization of bivalve morphology measurements.
(PNG)

S2 Fig PCs contribution to the shell shape. Contribution of the first five PCs to shape variation, with the average shell shapes, for both lateral and ventral views, represented for increasing values along each PC (-3 SD, Mean, +3 SD).
(JPEG)

S1 Table CT parameters. Computed tomography parameters (voltage, current, filter, voxel size, and ray detector) used for each mussel shell scanned.
(XLSX)

S2 Table Mussel shell statistics. Summary of which time periods and sites were statistically different from each other for density (micro density, bulk density, and apparent porosity), morphology (length: height ratio, length: width ratio and surface area: volume ratio), and thickness (% calcite, average thickness/ length and maximum thickness/ length). Different letters indicate which groups are statistically different to each other.
(XLSX)

S1 File Elliptical Fourier analysis code. R code for Elliptical Fourier analysis.
(DOCX)

S2 File Mixed effect model code. R code for the mixed effects model.
(DOCX)

S3 File PCA analysis code. R code for PCA analysis.
(DOCX)
