# Supplementary figures and images for: Using Museum collections to assess the impact of industrialization on mussel (Mytilus edulis) calcification
Source: PLoS One. 2024 Apr 17;19(4):e0301874. doi: 10.1371/journal.pone.0301874 (PMC11023280; doi:10.1371/journal.pone.0301874)

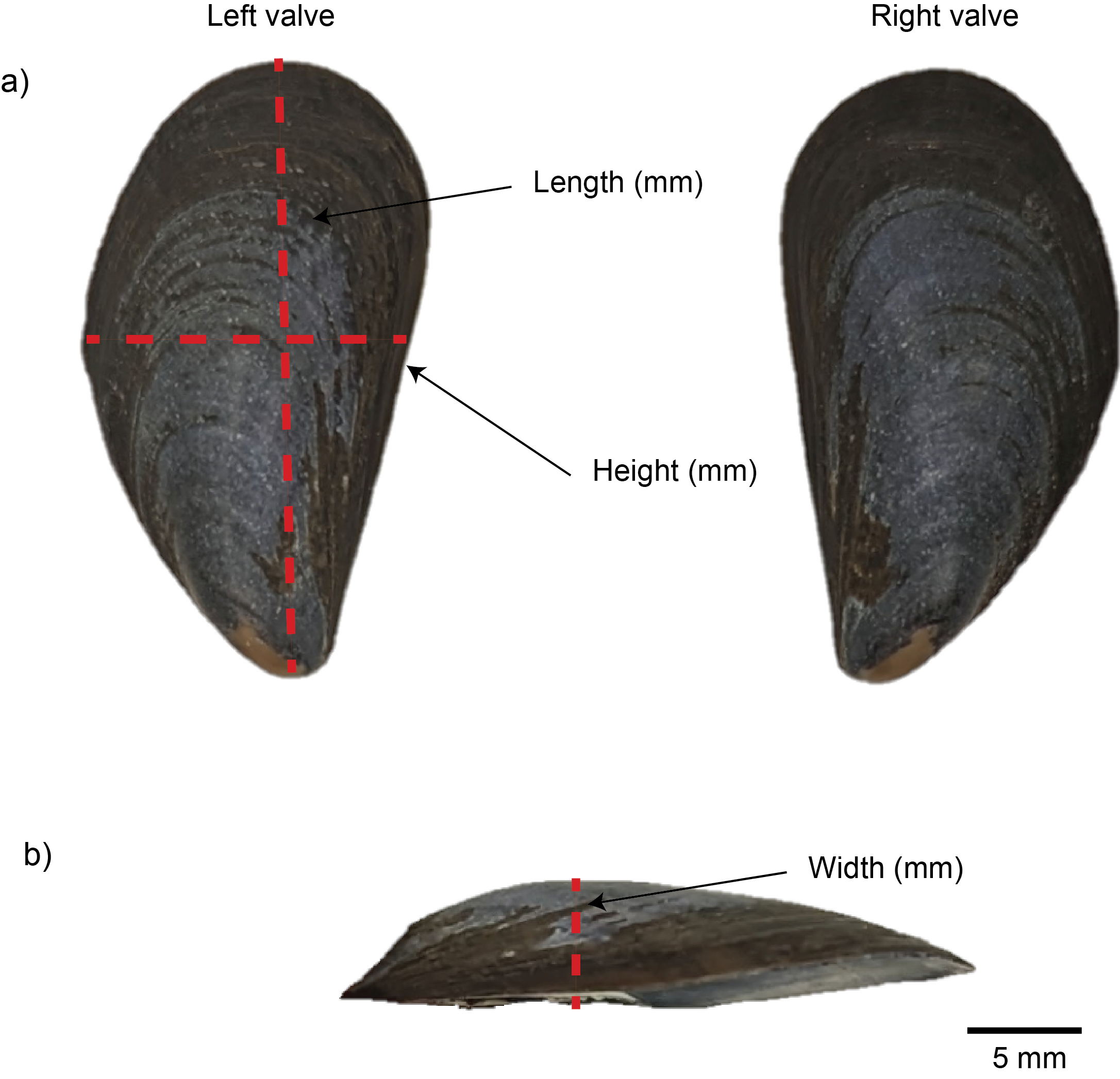

Supplement: S1 Fig — Visualization of bivalve morphology measurements. (PNG) [file pone.0301874.s001.png]

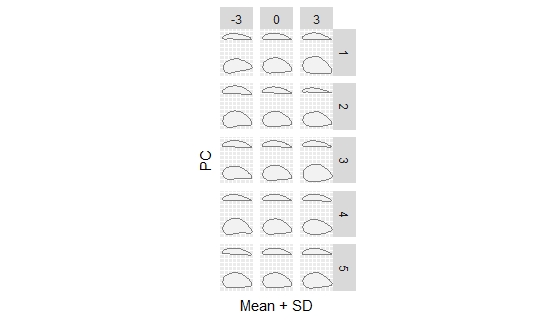

Supplement: S2 Fig — Contribution of the first five PCs to shape variation, with the average shell shapes, for both lateral and ventral views, represented for increasing values along each PC (-3 SD, Mean, +3 SD). (JPEG) [file pone.0301874.s002.jpeg]
