# Supplementary material for: Using Museum collections to assess the impact of industrialization on mussel (Mytilus edulis) calcification
Source: PLoS One. 2024 Apr 17;19(4):e0301874. doi: 10.1371/journal.pone.0301874 (PMC11023280; doi:10.1371/journal.pone.0301874)
Supplement: S1 File — R code for Elliptical Fourier analysis. (DOCX) [file pone.0301874.s005.docx]

S3: R code for Elliptical Fourier analysis.

*EFA morophometrics*

*library(RColorBrewer)*

*library(Momocs)*

*lf<-list.files('C:/Users/lmelbourne/OneDrive - AMNH/AMNH/Research/Bivalve study/EFA/images', full.names=TRUE)*

*shape<-import_jpg(lf)*

*fac<-lf_structure(lf, names=c("Image", "Year", "State",”Site”,"View", "Locality","Population", "Porosity", "SAV"), split = "_", trim.extension = TRUE)*

*mussel.shape<-Out(shape, fac = dplyr::tibble(fac))*

*panel(mussel.shape, fac="V3")*

*panel(mussel.shape, fac="V2")*

*panel(mussel.shape, fac="V4")*

*mussel.shape_v <- chop(mussel.shape, "View")*

*mussel.shape_v$L*

*mussel.shape_v$O*

*mussel.shape_v$O<-coo_smooth(mussel.shape_v$O,5)*

*mussel.shape_v$O<-coo_center(mussel.shape_v$O)*

*mussel.shape_v$O<-coo_scale(mussel.shape_v$O)*

*mussel.shape_v$O<-coo_sample(mussel.shape_v$O,1000)*

*mussel.shape_v$O<-fgProcrustes(mussel.shape_v$O)*

*mussel.shape_v$O<-coo_slidedirection(mussel.shape_v$O,"S")*

*stack(mussel.shape_v$O, xy.axis = F, title = NULL)*

*mussel.shape_v$L<-coo_smooth(mussel.shape_v$L,5)*

*mussel.shape_v$L<-coo_center(mussel.shape_v$L)*

*mussel.shape_v$L<-coo_scale(mussel.shape_v$L)*

*mussel.shape_v$L<-coo_sample(mussel.shape_v$L,1000)*

*mussel.shape_v$L<-fgProcrustes(mussel.shape_v$L)*

*mussel.shape_v$L<-coo_slidedirection(mussel.shape_v$L,"S")*

*stack(mussel.shape_v$L, xy.axis = F, title = NULL)*

*calibrate_reconstructions_efourier(coo_smooth(mussel.shape_v$O, 1000), range = 1:12)*

*calibrate_reconstructions_efourier(coo_smooth(mussel.shape_v$L, 1000), range = 1:12)*

*###determined to be 9 harmonics*

*calibrate_deviations_efourier(mussel.shape_v$O, id = 1:20, range = c(7,10,12,15))*

*calibrate_deviations_efourier(mussel.shape_v$L, id = 1:20, range = c(7,10,12,15))*

*###10 harmonics*

*calibrate_harmonicpower_efourier(mussel.shape_v$O, nb.h = 12)*

*calibrate_harmonicpower_efourier(mussel.shape_v$L, nb.h = 12)*

*###10*

*mussel.shape_v$O <- efourier(mussel.shape_v$O, nb.h = 10, norm = FALSE)*

*###Elliptical Fourier Analysis*

*mussel.shape.VO.coo.adj<-mussel.shape_v$O*

*mussel.shape.VL.coo.adj<-mussel.shape_v$L*

*mussel.shape.VO.coo.adj$fac$View<-droplevels(mussel.shape.VO.coo.adj$fac$View)*

*mussel.shape.VL.coo.adj$fac$View<-droplevels(mussel.shape.VL.coo.adj$fac$View)*

*mussel.shape.VO.coe<-mussel.shape.VO.coo.adj%>% efourier(nb.h = 10, norm = F)*

*mussel.shape.VL.coe<-mussel.shape.VO.coo.adj%>% efourier(nb.h = 10, norm = F)*

*mussel.shape_v$O <- efourier(mussel.shape_v$O, nb.h = 10, norm = FALSE)*

*mussel.shape_v$L <- efourier(mussel.shape_v$L, nb.h = 10, norm = FALSE)*

*mussel.shape.coe_v<-mussel.shape_v*

*mussel.shape.coe<-combine(mussel.shape_v)*

*hc1 <- hcontrib(mussel.shape_v$O, main = NULL, harm.r = 1:5, amp.r = c(0,1,2,3))*

*hc2 <- hcontrib(mussel.shape_v$L, main = NULL, harm.r = 1:5, amp.r = c(0,1,2,3))*

*###PCA*

*mussel.shape.pca<-PCA(mussel.shape.coe)*

*boxplot(mussel.shape.pca, fac = NULL, nax = 1:11)*

*scree_plot(mussel.shape.pca)*

*PC.contrib <- PCcontrib(mussel.shape.pca, nax = 1:5, sd.r = c(-3, 0, 3))*

*summary(mussel.shape.pca)*

*dev.new(width=10, height=10)*

*plot(mussel.shape.pca, fac = "Year", pos.shp = "full", nr.shp = 3, nc.shp = 3,*

*+ size.shp = 2, col.shp = "grey90", lwd.shp = 1.5, border = "grey40", nb.grids = 1,*

*+ cex.labelsgroups = 1.3, title = "", ellipsesax = F, ellipses = T, eigen = F,*

*+ conf.ellipses = 0.8, rug = F, col=brewer.pal(n=3, name="Dark2"), pch = 16, center.origin = FALSE)*

*dev.new(width=10, height=10)*

*plot(mussel.shape.pca, fac = "Site", pos.shp = "full", nr.shp = 3, nc.shp = 3,*

*+ size.shp = 2, col.shp = "grey90", lwd.shp = 1.5, border = "grey40", nb.grids = 1,*

*+ cex.labelsgroups = 1.3, title = "", ellipsesax = F, ellipses = T, eigen = F,*

*+ conf.ellipses = 0.8, rug = F, col=brewer.pal(n=3, name="Dark2"), pch = 16, center.origin = FALSE)*

*###Statistical analysis*

*mussel.shape.pc1 <- mussel.shape.pca$x[, 1]*

*mussel.shape.pc2 <- mussel.shape.pca$x[, 2]*

*mussel.shape.pc3 <- mussel.shape.pca$x[, 3]*

*mussel.shape.pc4 <- mussel.shape.pca$x[, 4]*

*mussel.shape.pc5 <- mussel.shape.pca$x[, 5]*

*mussel.shape.pc6 <- mussel.shape.pca$x[, 6]*

*mussel.shape.pc7 <- mussel.shape.pca$x[, 7]*

*mussel.shape.pc8 <- mussel.shape.pca$x[, 8]*

*mussel.shape.pc9 <- mussel.shape.pca$x[, 9]*

*mussel.shape.pc10 <- mussel.shape.pca$x[, 10]*

*mussel.shape.PCs <- data.frame(Year = mussel.shape.pca$fac$Year, State = mussel.shape.pca$fac$State, View = mussel.shape.pca$fac$View, Site = mussel.shape.pca$fac$Site, Locality = mussel.shape.pca$fac$Locality, Population = mussel.shape.pca$fac$Population, PC1=mussel.shape.pc1, PC2 = mussel.shape.pc2, PC3 = mussel.shape.pc3, PC4 = mussel.shape.pc4, PC5 = mussel.shape.pc5, PC6 = mussel.shape.pc6, PC7 = mussel.shape.pc7, PC8 = mussel.shape.pc8, PC9 = mussel.shape.pc9, PC10 = mussel.shape.pc10)*

*library(lattice)*

*library(gridExtra)*

*library(grid)*

*library(reshape2)*

*library(car)*

*library(nlme)*

*library(mgcv)*

*y <- mussel.shape.PCs[, 7:16]*

*Manova(lm(as.matrix(y) ~ Year + Site, data = mussel.shape.PCs), test.statistic = "Wilks")*
