# Supplementary material for: Using Museum collections to assess the impact of industrialization on mussel (Mytilus edulis) calcification
Source: PLoS One. 2024 Apr 17;19(4):e0301874. doi: 10.1371/journal.pone.0301874 (PMC11023280; doi:10.1371/journal.pone.0301874)
Supplement: S2 File — R code for the mixed effects model. (DOCX) [file pone.0301874.s006.docx]

S4: R code for the mixed effects model

*Mixed Effects model*

*require(car)*

*require (MASS)*

*require(tidyverse)*

*require(ggpubr)*

*require(rstatix)*

*require(broom)*

*require(lme4)*

*require(emmeans)*

*A$B.t<- A$B + 1*

*qqp(A$B.t, “norm) ###visual inspection for normality check, A = database, B = Parameter*

*f1<-lmer(B~Site+Year+(1|Individual), A)*

*### A = database*

*### B = morphological parameter*

*f2<-lmer(B~Site+(1|Individual), A) ## to focus on whether there are significant differences through time*

*f2<-lmer(B~Year+(1|Individual), A) ## to focus on whether there are significant differences between sites*

*anova(f1,f2)*

*Post hoc – pairwise comparison tests*

*pwc <- A %>% + group_by(Year) %>% + pairwise_t_test(B ~ Site, p.adjust.method = "bonferroni")*

*###identifying which sites are significant to each other within a time period*

*pwc2 <- A %>% + group_by(Site) %>% + pairwise_t_test(B ~ Year, paired = FALSE, p.adjust.method = "bonferroni" )*

*###identifying which time periods are significant to each other within a site*
