# Supplementary material for: Using Museum collections to assess the impact of industrialization on mussel (Mytilus edulis) calcification
Source: PLoS One. 2024 Apr 17;19(4):e0301874. doi: 10.1371/journal.pone.0301874 (PMC11023280; doi:10.1371/journal.pone.0301874)
Supplement: S3 File — R code for PCA analysis. (DOCX) [file pone.0301874.s007.docx]

S5: R code for PCA analysis

*code for PCA*

*library(RColorBrewer)*

*library(usethis)*

*library(devtools)*

*library(ggplot2)*

*library(plyr)*

*library(scales)*

*library(grid)*

*library(ggbiplot)*

*bivalve<-read.csv("A", header=TRUE) ### A =morphological database*

*bivalve.pca<-prcomp(bivalve[,c(6:10)], center=TRUE, scale=TRUE)*

*ggbiplot(bivalve.pca, ellipse=TRUE, groups=bivalve$Site) +*

*scale_color_brewer(palette = "Dark2")*

*ggbiplot(bivalve.pca, ellipse=TRUE, groups=bivalve$Year)+*

*scale_color_brewer(palette = "Dark2")*
